# PReS-FINAL-2041: Macrophage activation syndrome in the children with systemic juvenile idiopathic arthritis during the course of tocilizumab

**DOI:** 10.1186/1546-0096-11-S2-P54

**Published:** 2013-12-05

**Authors:** E Alekseeva, R Denisova, S Valieva, T Bzarova, K Isayeva, T Sleptsova, EA Mitenko, E Chistyakova, A Fetisova

**Affiliations:** 1 Rheumatology, Scientific Center Of Children's Health of RAMS, Moscow, Russian Federation

## Introduction

Systemic juvenile idiopathic arthritis (sjia) is a chronic inflammatory disease characterized by prolonged systemic and synovial inflammation. Life-threatening complication of sjia is the macrophage activation syndrome (MAS). Tocilizumab (TCZ) is highly effective in children with sjia. However, MAS remains under the treatment of TCZ.

## Objectives

To assess features of sjia developed macrophage activation syndrome during the course of TCZ therapy.

## Methods

122 patients (aged 1-17 years) with sjia received TCZ at the dose of 8 or 12 mg/kg every 2 or 4 weeks in Scientific Center of Children Health. Three patients (1 male-10 years, 2 females-7 and 1.9 years old) developed MAS during the course of TCZ treatment. In these patients, clinical features and laboratory findings were evaluated and compared with diagnostic guidelines.

## Results

The disease duration before initial infusion of TCZ was 8, 5,5 and 0,9 years. Two of them had history of MAS and developed MAS after first infusion of TCZ. One patient received corticosteroids at the onset of MAS - was 0.1 mg/kg. The diagnostic clinical criteria of MAS complicating sjia such as central nervous system dysfunction, hemorrhage were rarely observed. Furthermore, high fever was not seen at the onset of MAS in one patient. The laboratory criteria such as decreased platelet count, elevated levels of aspartate aminotransferase, LDH, decreased white blood cell count, and hypofibrinogenemia, hyperferritinemia were fulfilled in these patients. Additionally, ferritinemia was commonly observed in early phase of MAS. Since hyperferritinemia is hardly observed even in active s-JIA during TCZ treatment, it was useful to have early suspicion of MAS. However, one of the patients had normal level of CRP. One of them depeloped hepatitis with hyperbilirubinemia and high level of AST, ALT. After the diagnosis of MAS was established, infusion of metylprednisolone and prednisolone treatment per os (1-2 mg/kg/day) were immediately started, and the patients responded favorably to these treatments. The TCZ treatment was discontinued in two patients.

## Conclusion

MAS would occur unexpectedly despite no apparent trigger and few clinical symptoms during the course of TCZ. Blockade of IL-6 alone would not be sufficient to avoid occurrence of MAS. We should have early suspicion of MAS in case with thrombocytopenia and hyperferritinemia despite subtle clinical symptoms. Early therapeutic intervention would lead relatively good prognosis.

## Disclosure of interest

None declared.

